# Structure Validation of G‐Rich RNAs in Noncoding Regions of the Human Genome

**DOI:** 10.1002/cbic.201900696

**Published:** 2020-02-26

**Authors:** Oliver Binas, Irene Bessi, Harald Schwalbe

**Affiliations:** ^1^ Institute for Organic Chemistry and Chemical Biology Goethe University Frankfurt Max-von-Laue Strasse 7 60438 Frankfurt Germany; ^2^ Institute for Organic and Biomolecular Chemistry Julius Maximilians University Würzburg Am Hubland 97074 Würzburg Germany

**Keywords:** biophysical investigation, circular dichroism, G-quadruplexes, NMR spectroscopy, RNA

## Abstract

We present the rapid biophysical characterization of six previously reported putative G‐quadruplex‐forming RNAs from the 5′‐untranslated region (5′‐UTR) of silvestrol‐sensitive transcripts for investigation of their secondary structures. By NMR and CD spectroscopic analysis, we found that only a single sequence—[AGG]_2_[CGG]_2_C—folds into a single well‐defined G‐quadruplex structure. Sequences with longer poly‐G strands form unspecific aggregates, whereas CGG‐repeat‐containing sequences exhibit a temperature‐dependent equilibrium between a hairpin and a G‐quadruplex structure. The applied experimental strategy is fast and provides robust readout for G‐quadruplex‐forming capacities of RNA oligomers.

## Graphical abstract

**G‐quadruplex‐forming capacities of RNA oligomers**: We present the rapid biophysical characterization of six previously reported putative G‐quadruplex‐forming RNAs, from the 5′‐UTR of silvestrol‐sensitive transcripts, through investigation of their secondary structures by NMR and CD spectroscopic analysis.

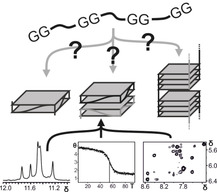

## Introduction

**Scheme 1:**
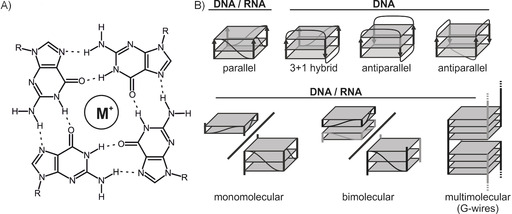
A) General chemical structure of a G‐tetrad featuring four guanine residues and a single monovalent cation (M^+^). B) Examples of the possible topologies featured in DNA and RNA G‐quadruplexes.

G‐quadruplexes are noncanonical inter‐ or intramolecular structural motifs formed by G‐rich DNA or RNA sequences. Their fundamental building blocks are planar tetrads composed of four guanine nucleobases (G‐tetrad) that engage in Hoogsteen‐type base pairing (Scheme 1 A).^1^, ^2^ Typically, two to four^3^ of these tetrads are stacked on top of each other to form the structural motif, though larger assemblies have been reported as well (Scheme 1 B).^4^, ^5^ G‐quadruplex folding requires monovalent cations, such as K^+^ or Na^+^, because the positive charge stabilizes the partially negatively charged O6 atoms of the guanine bases.^6^ Although DNA G‐quadruplexes show strong structural polymorphism due to loop variety, leading to various possible folding motifs,^7^ the presence of the 2′‐OH groups in RNA G‐quadruplexes favors a C3′‐*endo* sugar pucker and an all‐parallel folding topology (Scheme 1 B).^8^ The strong stacking interactions between the tetrads lead to a remarkable thermodynamic stability,^9^ with the stability of RNA G‐quadruplexes exceeding that of DNA G‐quadruplexes.^10^ DNA G‐quadruplexes can be detected in vivo^11^ and their fundamental role in telomere maintenance as well as in gene regulation is generally acknowledged.^12^ Bioinformatics and in vitro studies^11^, ^13^ suggested the presence of G‐rich putative quadruplex‐forming sites in untranslated regions (UTRs)^14^ of mRNAs, as well as in the transcripts of human telomers known as telomeric repeat‐containing RNA. RNA G‐quadruplexes might be involved in processes such as polyadenylation,^15^ modulation of translational efficiency,^16^, ^17^ and splicing.^18^ However, the existence of RNA G‐quadruplexes in vivo is a matter of current scientific debate.^19^ In 2016, in‐cell mapping experiments suggested that G‐rich cellular transcripts capable of forming G‐quadruplex structures after refolding in vitro are largely unfolded in eukaryotic cells.^20^ Later studies that combined RNA G‐quadruplex‐specific precipitation with sequencing were able to detect transient formation of RNA G‐quadruplex structures in human cells.^21^ Collectively, these findings suggest the existence of RNA G‐quadruplexes in vivo as dynamic structures, their folding/unfolding dynamics governed by the cellular machinery (possibly helicases).

By using high‐throughput probing methods, deep sequencing, and bioinformatics, several studies have identified G‐rich motifs that, it was speculated, might form G‐quadruplexes.^22^, ^23^, ^24^, ^25^ Moreover, investigation of the interaction between RNA G‐quadruplexes and protein counterparts often involves the use of short G‐rich oligomers^26^ that are assumed to form G‐quadruplexes in vitro, but without appropriate experimental evidence in support of this assumption. High abundances of guanosine residues in RNA transcripts can indicate G‐quadruplex formation, but structure, stability, and other biophysical properties remain unclear without detailed biophysical characterization of these G‐rich motifs. Although the RNA G‐quadruplex topologies are limited in terms of strand orientation by the strong preference of RNA to form all‐anti all‐parallel G‐quadruplexes, dimerization or multimerization can lead to a number of different general topologies that can alter biological function immensely (Scheme 1). While focusing primarily on the biological context and putting great emphasis on the role of G‐quadruplexes in regulatory systems, we wish to remark here that many cell biology studies do not involve any (or involve only sparse) biophysical characterization of G‐quadruplex structures.^27^, ^28^, ^29^, ^30^, ^31^

Herein, we show that such advanced biophysical structural characterization can be time‐ and cost‐effective and should be mandatory for any high‐profile study. It should be performed in order to establish whether putative G‐quadruplex‐forming sequences are indeed actually forming G‐quadruplex structures under the tested in vitro experimental conditions.

After an illustration of the general protocol for a preliminary biophysical screening, the analysis of six putative G‐quadruplex‐forming RNAs from the 5′‐UTR of silvestrol‐sensitive transcripts, previously reported in Nature, is presented in detail.^32^

### A simple biophysical protocol for validation of the formation of G‐quadruplex structures

We propose a stepwise screening method involving CD and NMR spectroscopic studies and incorporating increasingly sophisticated spectroscopic methods in each step, ultimately providing a strong dataset for the folding potential of RNA sequences predicted to form G‐quadruplex structures.

**Figure 1:**
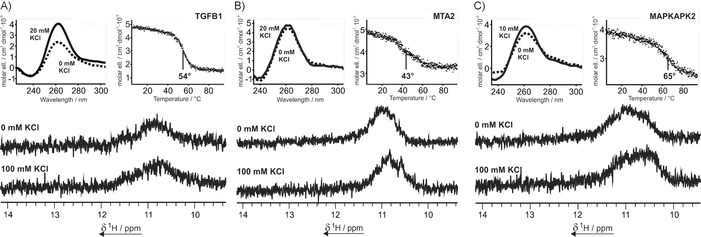
Top left in each panel: CD spectra of A) TGFB1, B) MTA2, and C) MAPKAPK2 in the presence of 0 mm KCl (—) and A), B) 20, or C) 10 mm KCl (‐ ‐ ‐ ‐). The solid line spectra mark the end point of a KCl titration (Figure S1). Samples contained 10 μm RNA and 10 mm BisTris**⋅**HCl buffer (pH 6.8). Top right in each panel: CD melting curves of A) TGFB1, B) MTA2, and C) MAPKAPK2 at 262 nm in the presence of 20, 20, and 10 mm KCl, respectively. Melting points were derived from sigmoidal fitting. Samples contained 10 μm RNA in 10 mm potassium phosphate buffer (pH 6.8). Bottom in each panel: imino region of the 1D ^1^H NMR spectrum of A) TGFB1, B) MTA2, and C) MAPKAPK2 in the presence of 0 mm and 100 mm KCl. Samples contained 100 μm RNA, 25 mm BisTris**⋅**HCl buffer (pH 6.8), and 10 % D_2_O in H_2_O. Severe peak broadening is observed due to the formation of large molecular aggregates.

In a first step, CD spectroscopy is used to determine the thermodynamic stabilities of the putative G‐quadruplex structures. Additionally, general information about the folding topology of the G‐quadruplex can be inferred from the CD signature (Figure 1).^33^ A strong response to addition of a monovalent cation (typically K^+^) is an indicator for G‐quadruplex formation; however, the formation of a different structure cannot be excluded unambiguously.

Although CD spectra are helpful for determining G‐quadruplex strand topology, the question of whether or not a G‐quadruplex is formed cannot be answered conclusively because the spectra of other structures such as G‐wires^34^ can look comparable or identical to those of G‐quadruplexes. 1D ^1^H NMR spectroscopy can help in identifying a G‐quadruplex unambiguously, because reporter signals of imino atoms involved in G‐tetrads resonate at a characteristic frequency of around 11 ppm. Other than determining the number of guanosine residues involved in the G‐quadruplex, 1D NMR data lack further structural information, but 2D NMR spectroscopy and, in particular, 2D ^1^H,^1^H NOESY can serve as tools for more advanced structural characterization in a third step.^35^ Essentially, 2D ^1^H,^1^H NOESY spectra contain all distances below a threshold of ≈6 Å between the protons of the oligonucleotide. Although these data might even be sufficient for the calculation of a basic 3D structure, the assignment of all protons can be tedious and time‐consuming and requires data from other 2D NMR methods. Even without an in‐depth analysis, however, the general structural features of a G‐quadruplex can be determined by analysis of 2D ^1^H,^1^H NOESY spectra. Further, NMR‐spectroscopic methods such as DOSY^36^ or heterocorrelated NMR can yield additional structural information.

By following these increasingly complex steps, it is possible to achieve the biophysical and structural characterization of any small G‐quadruplex‐forming oligonucleotide. It should be noted that CD and NMR measurements require 0.05–0.5 mm samples in a volume of approx. 0.5 mL, a quantity that can easily be ordered and delivered within only a few working days at comparatively low cost.

### A case study—silvestrol‐sensitive G‐rich transcripts from human 5′‐UTR

Recent studies have suggested the involvement of G‐quadruplexes in the function of the anticancer therapeutic silvestrol,^37^ which inhibits the initiation factor eIF4A in human T‐ALL‐infected cell lines.^38^ Wendel et al. found an accumulation of G‐rich sequences in the 5′‐UTR of human mRNA that experienced downregulation of the translational efficiency under the influence of silvestrol.^32^ Several G‐rich sequential motifs that showed a CD profile indicative of G‐quadruplex formation were identified.

In this study, we have characterized the tendency of these six short putatively G‐quadruplex‐forming RNAs from the 5′‐UTR of silvestrol‐sensitive mRNA transcripts to undergo G‐quadruplex formation. By using the protocol described above, including NMR spectroscopy, we were able to monitor directly the secondary structures actually formed and to assess the influence of varying conditions, such as K^+^, concentration, and temperature. Five of the G‐rich RNA sequences, screened in this work (Table 1), are among the most silvestrol‐sensitive RNA transcripts, as determined in studies by Wendel et al.^32^ In addition, two flanking U residues were added to EP300 to assess their effect on the overall structure and to impede G‐quadruplex stacking,^39^ resulting in the sequence UEP300U.

**Table 1:** RNA sequences screened throughout the study and the corresponding effects of KCl addition as observed by CD and NMR spectroscopy.

Name	Sequence	Length	Effect of KCl addition	Effect of KCl addition	Type of NMR
		[nt]	(CD signal at 260 nm)	(NMR signal 10–12 ppm)	signals observed
TGFB1	5′‐GGGAGGAGGGGGA‐3′	13	moderate increase	none observed	broad bulge
MTA2	5′‐GGGGGCGGGGGUA‐3′	13	none observed	slight upfield shift	broad bulge
MAPKAPK2	5′‐GGGGGGCGGCGGG‐3′	13	minor increase	slight upfield shift	broad bulge
ADAM10	5′‐AGGAGGCGGCGGC‐3′	13	strong increase	signals appear	defined imino signals
EP300	5′‐CGGCGGCGGCGG‐3′	12	minor increase	temperature‐dependent chemical shift changes	defined imino signals
UEP300U	5′‐UCGGCGGCGGCGGU‐3′	14	minor increase	temperature‐dependent chemical shift changes	defined imino signals

CD spectroscopic examination of TGFB1, MTA2, and MAPKAPK2 showed a profile indicative of an all‐parallel G‐quadruplex structure^40^, ^41^ with a maximum at 262 nm and a minimum at 240 nm at 20 mm, 20 mm, and 10 mm KCl, respectively (Figure 1, top left in each panel). No spectral changes were visible after addition of more K^+^ ions to the system (Figure S1 in the Supporting Information). Even without K^+^ ions, a maximum in ellipticity at 262 nm was already observable. G‐tetrad formation requires monovalent cations, so this is atypical for a G‐quadruplex and hints at the formation of a different secondary structure. The melting points could be determined by CD melting curve analysis (Figure 1, top right in each panel) and were determined as 54, 43, and 65 °C for TGFB1, MTA2, and MAPKAPK2, respectively.

The type of secondary structure was further investigated by using NMR spectroscopy. The 1D ^1^H spectra of all three sequences showed only a very broad signal in the imino region between 10 and 12 ppm (Figure 1, bottom panels). This region is typical for Hoogsteen‐paired residues as observed in G‐quadruplexes.^42^ Although G‐quadruplexes, as compact structures, show distinct peaks in the imino region in 1D ^1^H NMR spectra, we assume that these G‐rich RNA sequences form a higher‐order polymorphic structure. The NMR data suggest the formation of high‐order unspecific aggregates, because large structures lead to broadening of peaks in NMR spectra, while the peak positions differ throughout the numerous different possible lengths of such structures.^43^ These aggregates might interact through GG N1‐carbonyl symmetric base pairs, which can be formed even in the absence of monovalent cations, as recently shown by Plavec et al.^44^ Addition of KCl did not lead to any observable change in the NMR spectra, whereas the CD spectra, at least in the case of TGFB1, showed that a rearrangement takes place. The strong positive signal at 262 nm is also in agreement with the formation of G‐wires,^5^ as observed by Protozanova and Macgregor.^34^ For larger aggregates of those G‐wires, the detection of a broad signal in 1D ^1^H NMR spectra is expected.^45^ In native PAGE experiments the bands of TGFB1 and MAPKAPK2 are strongly broadened, thus supporting the proposed folding scheme of highly polymorphic structures (Figure S2). MTA2 shows, apart from the same broad bands, a small defined band with an intensity that diminishes upon refolding (Figure S2).

**Figure 2:**
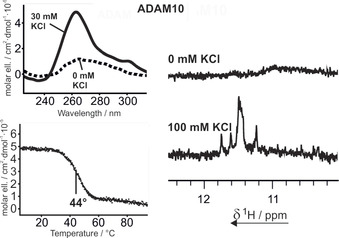
Top left: CD spectrum of ADAM10 in the presence of 0 (‐ ‐ ‐ ‐) and 30 mm (—) KCl; 30 mm KCl marked the endpoint of the titration (Figure S1). The sample contained 10 μm RNA and 10 mm BisTris**⋅**HCl buffer (pH 6.8). Bottom left: CD melting curve of ADAM10 at 262 nm in the presence of 30 mm KCl. The melting point was derived from sigmoidal fitting. The sample contained 10 μm RNA in 10 mm potassium phosphate buffer (pH 6.8). Right: Imino region of the 1D ^1^H NMR spectrum of ADAM10 in the presence of 0 and 100 mm KCl. Samples contained 100 μm RNA, 25 mm BisTris**⋅**HCl buffer (pH 6.8), and 10 % D_2_O in H_2_O.

ADAM10 showed no significant circular dichroism signal without the addition of KCl (Figure 2, top left). This contrasts with the CD data for TGFB1, MTA2, and MAPKAPK2 (Figure 1), because those sequences showed strong signals at around 260 nm even without addition of KCl. When, however, KCl was added to a sample of ADAM10, a positive ellipticity at 260 nm could be observed. The end point of the titration was reached at 30 mm KCl, and no further increase in ellipticity could be measured. CD melting analysis was thus carried out at a KCl concentration of 30 mm. The melting curve (Figure 2, bottom left) shows a clear sigmoidal profile, and the melting point could be determined as 44 °C.

G‐quadruplex formation could be confirmed by 1D ^1^H NMR data (Figure 2, right). In the absence of KCl, no signal apart from a small broadened bulge was observed in the imino region. This hints at the formation of unspecific aggregates, analogously to the cases of TGFB1, MTA2, and MAPKAPK2. After KCl had been added, however, three sharp separated resonances and several overlapped signals at 11.2–11.3 ppm could be detected. Eight signals hint at a stack of two G‐tetrads, as can be expected from the sequence [AGG]_2_[CGG]_2_C.

**Figure 3:**
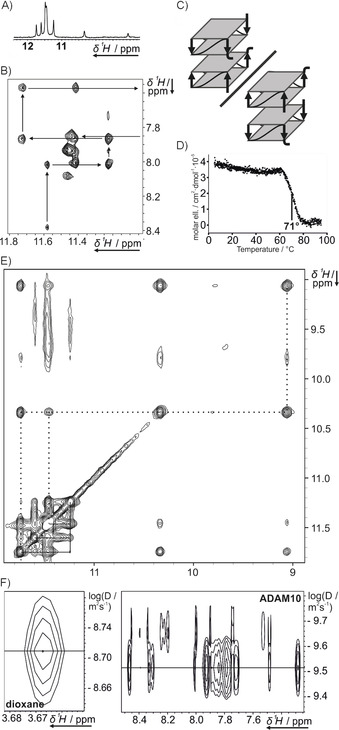
Further investigation of ADAM10 by 2D NMR spectroscopy. A) Imino region of the 1D ^1^H spectrum. B) Imino–aromatic region, highlighting strand interactions. C) Four‐tetrad G‐quadruplex layout as suggested from 2D data. D) CD melting curve at an RNA concentration of 300 μm. E) Full imino region of the 2D ^1^H,^1^H NOESY spectrum, highlighting two peaks showing three NOESY crosspeaks. F) DOSY of ADAM10 showing zooms of the 1,4‐dioxane reference peak and the aromatic RNA peaks.

The formation of a highly symmetric dimer, though, cannot be ruled out by 1D ^1^H NMR spectroscopy alone.^46^ We acquired 1D ^1^H and 2D ^1^H,^1^H NOESY spectra at a higher concentration (700 μm) and discovered two additional imino proton signals, resonating at 10.3 and 9.3 ppm (Figure 3 A), that were only faintly visible at 50 μm. A possible hypothesis that could explain this strong chemical shift perturbation might be ring current effects due to the stacking of terminal or loop residues on the G‐tetrad. We investigated the general topology of the G‐quadruplex by analysis of the ^1^H,^1^H NOESY spectrum (Figure 3 B, E). On inspection of the imino proton crosspeaks, we found that crosspeaks from imino proton signals to three other signals are visible (Figure 3 E, solid and dashed lines); this would not be expected from a two‐tetrad G‐quadruplex but could be explained by these residues lying between two tetrads, as in three‐ or four‐tetrad G‐quadruplexes. In this case two imino proton crosspeaks to the adjacent tetrads and a third crosspeak to the neighboring base in the same tetrad would explain the observation. Additionally, the imino to aromatic crosspeak region shows that it is possible to follow cross‐signals over more than two H1–H8 layers (Figure 3 B).

From these observations, the topology of the ADAM10 G‐quadruplex would be expected to include four G‐tetrads, so a bimolecular G‐quadruplex should be formed. We were able to confirm the formation of such a higher‐order structure by DOSY spectroscopy (Figure 3 F), in which we determined a hydrodynamic radius of 13.7 Å. A comparable value of 13.9 Å was observed in the four‐tetrad G‐quadruplex of the modified DNA GG‐Az1‐GG.^46^ The DOSY peaks of ADAM10 and of the reference substance 1,4‐dioxane are shown in Figure 3 F. Additional peaks with a higher diffusion coefficient are observed, corresponding to an even higher‐order structure with a hydrodynamic radius of 18.8 Å that is partially populated.

To provide additional verification of the formation of dimerization we conducted CD melting experiments at a higher concentration (300 μm, Figure 3 D). We observed a rise in the melting temperature from 44 to 71 °C, in comparison with the lower concentration (10 μm) investigated before (Figure 2). From the number of imino peaks observed, symmetry can be proposed. Combined with the requirement of an all‐anti all‐parallel G‐quadruplex, this leads to two possible topologies (Figure 3 C). These differ in the stacking tetrads, which feature the 5′‐ or 3′‐terminal residues.^47^ The exact determination of the topology including the G‐residue polarity would require a full assignment of the NMR signals. Only in rare cases can this be achieved from NOESY data alone; most commonly it involves isotopic labeling of single nucleotides in various NMR samples^48^ or uniform labeling of one NMR sample. These techniques require considerable preparative effort, so their application is beyond the scope of a topological screening, as carried out here.

**Figure 4:**
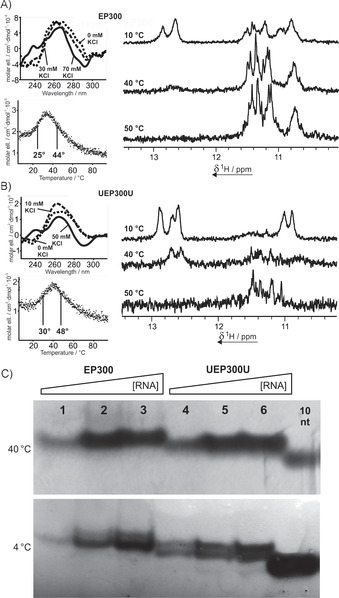
Top left: CD spectra of A) EP300, and B) UEP300U in the presence of 0 mm (dotted lines), A) 30 mm, or B) 10 mm (dashed lines), and A) 50 mm, or B) 70 mm KCl (solid lines). Concentrations of 50 and 70 mm KCl marked the end points of the titration. The sample contained 10 μm RNA and 10 mm BisTris**⋅**HCl buffer (pH  6.8). A), B) Bottom left: CD melting curves of EP300 and UEP300U at 262 nm in the presence of 50 or 70 mm KCl, respectively. Melting points were derived from sigmoidal fitting. The sample contained 10 μm RNA in 10 mm potassium phosphate buffer (pH 6.8). Right: Imino regions of the 1D ^1^H NMR spectra of EP300 and UEP300U at 10, 40, and 50 °C. Samples contained 100 μm RNA, 25 mm BisTris**⋅**HCl buffer (pH 6.8), and 10 % D_2_O in H_2_O. C) Native PAGE (15 %) of EP300 and UEP300U at 4 and 40 °C at increasing RNA concentrations. The gel was run for 4 h at 0.5 W with 1× TBE buffer (50 mm KCl) as running buffer. Samples contained 12, 24, and 36 μm (EP300) or 20, 40, and 60 μm (UEP300U) RNA, 30 % glycerol, 50 mm KCl, and 1× TBE buffer. 10 nt ssRNA was used as a reference.

In circular dichroism spectra of EP300 (Figure 4 A, top left) only moderate changes in ellipticity were visible upon addition of KCl. Whereas the peak at 260 nm only showed slight variation in intensity, a small positive peak at 240 nm and a negative peak at 290 nm appeared at high KCl concentrations. Subsequently, circular dichroism at 260 nm was measured over a temperature range between 5 and 95 °C, revealing biphasic behavior with two different transition points: 25 and 44 °C. Subsequent analysis of the NMR imino proton region on titration with KCl, as well as of its temperature dependence, led to the same finding. At 0 °C signals with equal intensity could be observed in the regions characteristic of Hoogsteen base pairs (10–12 ppm) and of Watson–Crick base pairs (12–14 ppm). Upon heating, the low‐field signals diminished, vanishing completely above 30 °C. Simultaneously, the intensity of the high‐field imino signals increased. This observation indicates a thermal equilibrium between a duplex or hairpin and a G‐quadruplex, with the G‐quadruplex showing a higher thermal stability. The two states showed different electrophoretic mobilities in native PAGE, resulting in two slightly separated bands at 4 °C but in no such observation being made at 40 °C (Figure 4, lanes 1–3). The electrophoretic mobility of the duplex or hairpin structure is comparable with that observed for the G‐quadruplex structure.

The same set of experiments was conducted on UEP300U, with comparable results. Interestingly, addition of two flanking U residues to the sequences enhances the thermal stability of the low‐temperature structure (possibly duplex or hairpin) relative to that of the G‐quadruplex. Thus, imino signals of Watson–Crick bound residues were observed even at 40 °C, whereas in the case of EP300 these signals had completely vanished at this temperature. Analysis of the CD melting curve supports this observation, with the melting point of the low‐temperature structure being shifted to 30 °C for UEP300U. The CD profiles of UEP300U showed a more pronounced potassium dependency then those of EP300. The spectral changes upon addition of small amounts of KCl were subtle and led to a spectrum characteristic of an all‐anti all‐parallel quadruplex, whereas at higher concentrations a positive signal at 240 nm and a negative signal at 290 nm were observed, accompanied by a decrease in the positive signal at 260 nm. Such spectra have previously also been observed in the cases of Z‐DNA^49^ and Z‐RNA.^50^ These helices can form from purine‐pyrimidine repeat sequences.^51^ Hairpin formation with two [CG] base pairs and a GG mismatch has been observed previously,^52^ so formation of a duplex mimicking the CD characteristics of Z‐RNA at high salt concentrations could explain the unusual CD spectral characteristics of EP300 and UEP300U. In contrast, Rypniewski et al. observed an A‐type helical structure of CGG repeats in crystallographic studies.^53^ As in the case of EP300, the two structures of UEP300U at low temperature can also be observed in native PAGE (Figure 4, lanes 4–6).

## Discussion

Herein, we report a three‐step protocol for the characterization of putative G‐quadruplex‐forming RNA oligomers. The protocol involves CD and NMR screening, including CD melting curve analysis and 1D and 2D NMR.

We have demonstrated the application of the protocol by using NMR and circular dichroism studies to probe the structural preferences of six different G‐rich RNAs—from the 5′‐UTR of human mRNA—that are involved in the modulation of eIF4A suppression by silvestrol. It was found that three of the sequences, despite being G‐rich, did not form distinct G‐quadruplex structures, but produced aggregates in an unspecific manner. The [XGG]_4_ repeat sequences ADAM10, EP300, and UEP300U, however, did fold into G‐quadruplexes, with EP300 and UEP300U existing in thermal equilibrium with hairpin structures.

Circular dichroism studies of four of the putative G‐quadruplex‐forming sequences showed high spectral similarities, especially under the influence of KCl. CD spectra of TGFB1, MTA2, MAPKAPK2, and ADAM10 each showed a strong positive band at 260 nm after addition of KCl, with differences being merely visible before the addition, when the RNA was assumed to be unfolded. Before KCl addition, MTA2, MAPKAPK2, and—to some extent—TGFB1 already showed a positive peak. By using NMR spectroscopy and PAGE we were only able to confirm G‐quadruplex folding in the case of ADAM10, which forms a four‐layer quadruplex. This quadruplex shows a low thermal stability with a melting point of only 44 °C at a concentration of 10 μm. At 300 μm this rises to 71 °C, likely due to dimer formation, which leads to a four‐tetrad structure. These have already been observed to be thermodynamically more stable than two‐tetrad G‐quadruplexes.^54^ The bimolecular nature of this quadruplex is only evident through 2D NMR investigation but could be confirmed by DOSY NMR spectroscopy. NMR spectra revealed that TGFB1, MTA2, and MAPKAPK2 form unspecific aggregates of high molecularity with or without addition of KCl. The absence of a monovalent cation hinders G‐tetrad formation, so interaction through GG N1‐carbonyl symmetric base pairs is assumed in this case. Under the influence of potassium, a change in the CD spectrum of TGFB1 suggests the formation of G‐wires, whereas the behavior of MTA2 and MAPKAPK2 remains unclear. Because NMR spectroscopy fails to resolve structures of such a size, those structural preferences cannot be confirmed beyond doubt, but G‐quadruplex formation can be ruled out. The structural polymorphism is supported by native PAGE, with the gel bands being broadened almost beyond detection. The small defined band of MTA2, which is observed alongside the broad main band, diminishes upon refolding, thus indicating a small thermodynamic barrier to aggregate formation.

The structure of CGG‐repeat‐containing RNA is a subject of current scientific debate.^55^, ^56^ Contributing to this, we collected CD and NMR spectroscopic data for the sequences [CGG]_4_ (EP300) and U[CGG]_4_U (UEP300U). CGG repeats are known to form helical structures with a non‐Watson–Crick GG base pair.^52^ We were able to identify Watson–Crick bases in the NMR spectra of EP300 and UEP300U accordingly. Additionally a high‐temperature structure exists in a temperature‐dependent structural equilibrium. It is assumed to be a G‐quadruplex because the involved imino protons resonate in the region typical of Hoogsteen‐bound G residues. Although the temperature‐dependent transition between two structural states was determined by CD melting studies, the structural characteristics of each state could be deconvoluted only by NMR. We were able to show that high temperature favors the formation of the G‐quadruplex over the duplex. The transition temperature from hairpin to G‐quadruplex was higher in the case of the uridine‐flanked sequence UEP300U. This might be because of the capping effect of dangling ends, which stabilizes RNA duplexes.^57^ In addition, flanking sequences can negatively affect the stability of G‐quadruplex structures;^58^ this adds to the higher relative stability of the hairpin. Even though G‐quadruplexes are stabilized by monovalent cations, the duplex seems to be the preferred conformation at high salt concentrations, if room temperature data as observed from CD experiments are considered. Despite CGG repeats forming an A‐helix in crystals and Z‐RNA in general forming at very high salt concentrations,^59^ CD spectra of EP300 and UEP300U hint at duplexes that exhibit characteristics of Z‐RNA. The reason for this behavior remains unclear. However, such structural equilibria are of great biological importance and can be tuned by cellular key factors such as cation levels^60^ or tRNA concentration.^61^ The electrophoretic mobility of the duplex structure is slightly lower than the mobility of the G‐quadruplex in both RNAs. Because the electrophoretic mobility in native PAGE depends not only on size, but also on compactness of the structure, the molecularity cannot directly be deduced from this data point. NMR spectra of the duplex show at least five imino signals, thus hinting at a bimolecular duplex.

## Conclusions

We were able to elucidate the conformational space of six small G‐rich mRNA fragments, from human 5′‐UTR of mRNA transcripts that are sensitive to silvestrol, by applying a three‐step screening protocol involving CD and NMR spectroscopy. Three of the six oligonucleotides do not fold into G‐quadruplexes as expected, but instead aggregate unspecifically. ADAM10 ([AGG]_2_[CGG]_2_C) forms a four‐tetrad all‐anti all‐parallel G‐quadruplex. EP300 and UEP300U ([CGG]_4_ and U[CGG]_4_U) each fold either into a duplex or into a G‐quadruplex depending on the conditions, in particular temperature and salt concentration. G‐quadruplex structures are believed to be involved in the therapeutic mechanisms of the anticancer drug silvestrol, so understanding of the structural characteristics of the investigated G‐rich sequences is of great importance for discriminating between potential G‐quadruplexes and other G‐rich sequences. By applying the three steps of our screening protocol in order, we were able to show that CD spectroscopy, although offering a rapid and cost‐effective method for obtaining preliminary information on the nature of a secondary structure, cannot be used alone to assess the actual conformation of a G‐rich oligonucleotide. NMR spectroscopy is necessary to unravel the information obtained by CD spectroscopy and to shed light on the actual conformation(s) present in solution. The protocol could be useful for obtaining all data points necessary for characterization of the structural characteristics of such oligonucleotides.

## Experimental Section

**RNA sample preparation**: The oligoribonucleotide sequences [GGGAGGAGGGGGA] (TGFB1), [GGGGGCGGGGGUA] (MTA2), [GGGGGGCGGCGGG] (MAPKAPK2), [AGG]_2_[CGG]_2_C (ADAM10), [CGG]_4_ (EP300), and U[CGG]_4_U (UEP300U) were bought from Dharmacon (GE Healthcare). RNA samples were purified (HPLC), desalted, precipitated with LiClO_4_ (2 %, *w*/*v* in acetone, 5 volumes) and stored in aqueous stock solutions.

**Circular dichroism**: All CD experiments were carried out with a Jasco J‐810 CD spectrometer (Jasco, GmbH) and use of quartz optical cuvettes with 0.1 cm path length (0.01 cm path length was used for ADAM10 at 300 μm concentration). For CD titration experiments with KCl, RNA samples with a concentration of 10 μm in potassium‐free BisTris**⋅**HCl buffer (pH 6.8, 10 mm) were prepared. The titration range was 0 to 70 mm KCl. Resulting CD spectra were baseline‐corrected and corrected for sample dilution. The data were smoothed by application of a Savitzky–Golay filter^62^ (10 points).

For CD melting curves the samples contained the RNA of interest (10 μm, 300 μm in one additional experiment for ADAM10), potassium phosphate buffer (pH 6.8, 10 mm), and potassium chloride according to the previously determined end point of the titration. The peak ellipticity was monitored over a temperature range from 5 to 95 °C with a scan rate of 1 °C min^−1^.

**Native PAGE**: Aqueous stock solutions of RNA were diluted to concentrations of 10–30 μm in TBE buffer (1×) with addition of glycerol (30 %) and KCl (50 mm). Polyacrylamide gels (15 %) containing TBE (1×) and KCl (50 mm) were cast and run with the samples at 0.5 to 0.8 W for 4 h at 4 or 40 °C. The running buffer contained TBE (1×) and KCl (50 mm). Gel visualization was achieved by GelRed staining and subsequent imaging under UV light.

**Nuclear magnetic resonance**: 1D ^1^H NMR spectra were acquired with 800 MHz Bruker AVIII (MTA2, MAPKAPK2), 600 MHz Bruker AVIII HD (TGFB1, ADAM10), and 600 MHz Bruker AVII (EP300, UEP300U) spectrometers (Bruker Biospin) equipped with Cryo TCI ^1^H[^13^C,^15^N], Prodigy TCI ^1^H[^13^C,^15^N], and Cryo TCI ^1^H[^13^C,^15^N] probes, respectively. Samples contained RNA (100 μm), BisTris**⋅**HCl (pH 6.8, 25 mm) for KCl titrations or potassium chloride buffer (pH 6.8, 25 mm) for temperature series, and DSS (25 μm) in H_2_O/D_2_O (9:1, *v*/*v*). The final sample volumes of 280 μL were transferred into 5 mm Shigemi tubes (Shigemi, Inc.). 1D ^1^H NMR spectra were recorded with 256 scans, 4096 points, and 1.5 s relaxation delay. Water suppression was applied by using a jump‐and‐return echo sequence.^63^

## Conflict of interest

*The authors declare no conflict of interest*.

## Supporting information

As a service to our authors and readers, this journal provides supporting information supplied by the authors. Such materials are peer reviewed and may be re‐organized for online delivery, but are not copy‐edited or typeset. Technical support issues arising from supporting information (other than missing files) should be addressed to the authors.

Supplementary
