# Supplementary material for: Structure Validation of G‐Rich RNAs in Noncoding Regions of the Human Genome
Source: Chembiochem. 2020 Feb 26;21(11):1656–63. doi: 10.1002/cbic.201900696 (PMC7318348; doi:10.1002/cbic.201900696)
Supplement: Supplementary file 1 — Supplementary [file CBIC-21-1656-s001.pdf]

CHEMBIOCHEM

Supporting Information

Structure Validation of G-Rich RNAs in Noncoding Regions of the Human Genome

Oliver Binas,[a] Irene Bessi,[b] and Harald Schwalbe*[a]

cbic_201900696_sm_miscellaneous_information.pdf

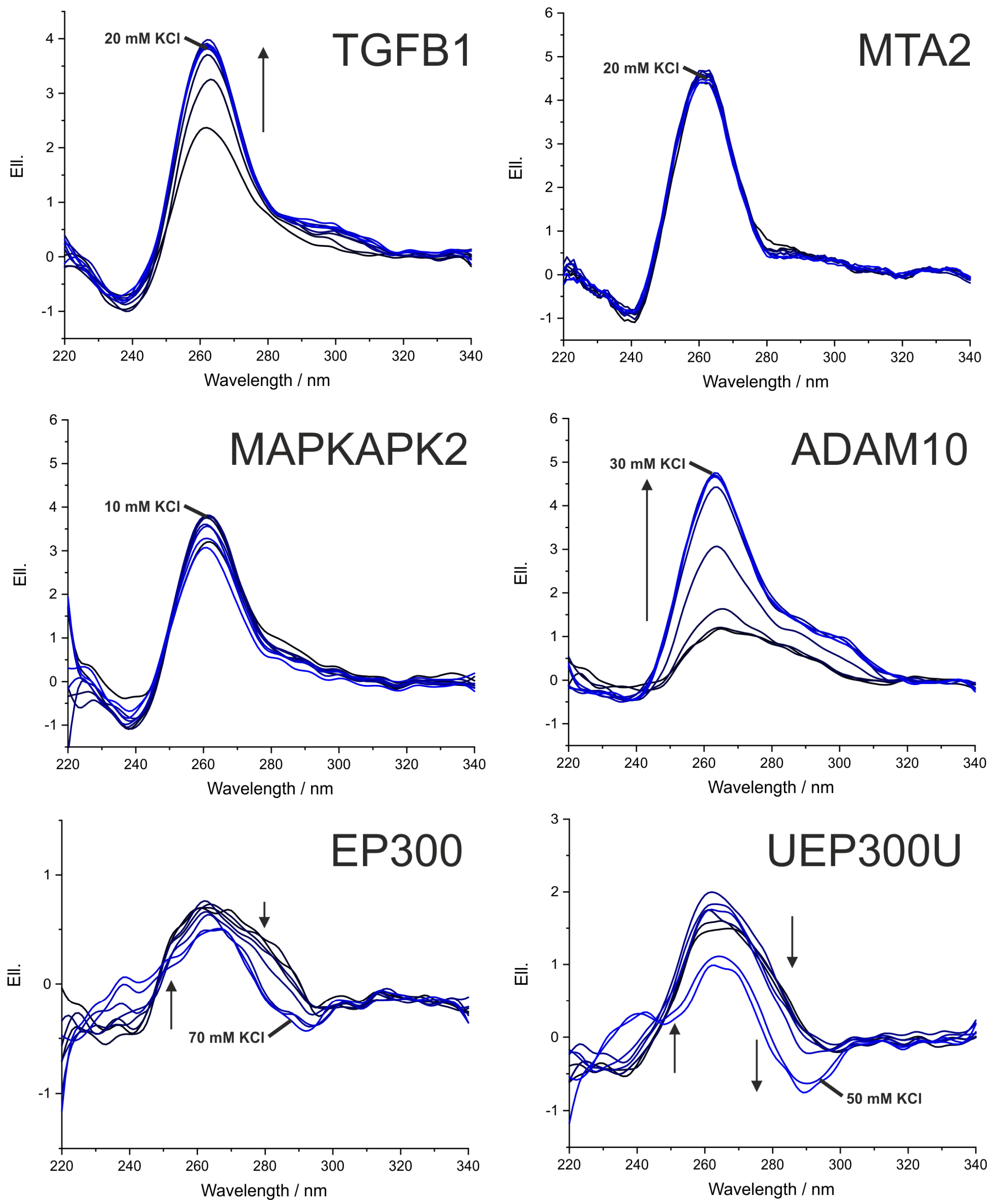

Figure S1. Raw CD KCl titration data of investigated RNAs. CD curves are colored from black (0 mM KCl) to blue (70 mM KCl). Arrows indicate changes in the CD signatures observed at higher KCl concentration. Endpoints of the titration mentioned in the manuscript are marked.

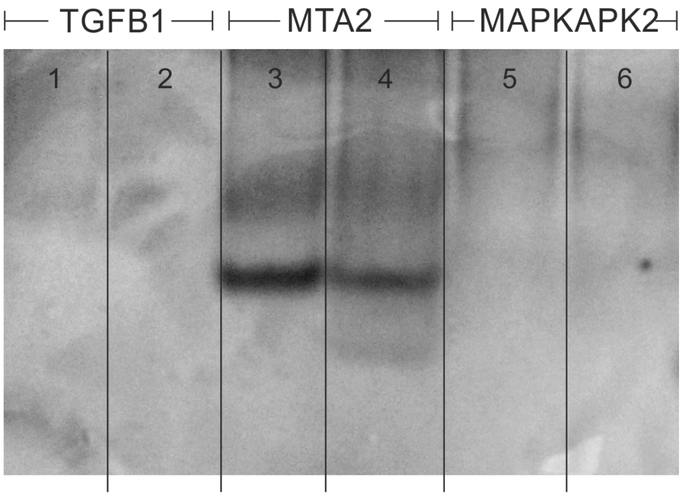

Figure S2. 15%-Native PAGE of TGFB1, MTA2 and MAPKAPK2. Without refolding (lanes 1, 3, 5) and after refolding by heating to 95 °C and rapid cooling (lanes 2, 4, 6).
